# A CCD-based reader combined with CdS quantum dot-labeled lateral flow strips for ultrasensitive quantitative detection of CagA

**DOI:** 10.1186/1556-276X-9-57

**Published:** 2014-02-04

**Authors:** Chen Gui, Kan Wang, Chao Li, Xuan Dai, Daxiang Cui

**Affiliations:** 1 Institute of Nano Biomedicine and Engineering, Key Laboratory for Thin Film and Microfabrication of Ministry of Education, Institute of Micro/Nano Science and Technology, Shanghai Jiao Tong University, 800Dongchuan Road, Shanghai 200240, People's Republic of China; 2 Research Institute of Translation Medicine, Shanghai Jiao Tong University, 800Dongchuan Road, Shanghai 200240, People's Republic of China

**Keywords:** Test strip reader, CCD, Revised WTHE algorithm, CdS quantum dots, CagA

## Abstract

Immunochromatographic assays are widely used to detect many analytes. CagA is proved to be associated closely with initiation of gastric carcinoma. Here, we reported that a charge-coupled device (CCD)-based test strip reader combined with CdS quantum dot-labeled lateral flow strips for quantitative detection of CagA was developed, which used 365-nm ultraviolet LED as the excitation light source, and captured the test strip images through an acquisition module. Then, the captured image was transferred to the computer and was processed by a software system. A revised weighted threshold histogram equalization (WTHE) image processing algorithm was applied to analyze the result. CdS quantum dot-labeled lateral flow strips for detection of CagA were prepared. One hundred sera samples from clinical patients with gastric cancer and healthy people were prepared for detection, which demonstrated that the device could realize rapid, stable, and point-of-care detection, with a sensitivity of 20 pg/mL.

## Background

Up to date, lateral flow tests, also called lateral flow immunochromatographic assays, have been widely used in qualitative and semiquantitative detection of biomarkers. This technology utilizes antigen-antibody reaction features to detect numbers of analytes, including antigens, antibodies, and even the products of nucleic acid amplification tests [1,2]. They have merits of user-friendly format, rapid detection, long-term stability, and relatively low cost [3,4]. However, most colloidal gold lateral flow tests are analyzed by naked eyes, which is subjective and inaccurate. For these reasons, many groups have engaged in developing novel labeling materials to replace colloidal gold. Quantum dots (QDs), one kind of novel nanomaterial, are composed of periodic groups of II-IV, III-V, or IV-VI semiconductor material. Quantum dots have many advantages compared with colloidal gold, such as high fluorescent intensity, broad absorption spectra, narrow and symmetric emission bands, and excellent stability [5-12]. On the other hand, most lateral flow tests could only implement qualitative detection. In order to realize quantitative detection, some groups [13-17] have dedicated to this issue. Huang et al. [2] utilized a photomultiplier tube (PMT) as a signal acquisition device for up-conversion of nanoparticle-labeled test strips. Although PMT has high sensitivity, it is with a limited surveyed area. Mei's group [1] chose a complementary metal oxide semiconductor (CMOS) image sensor to capture test strip images. Besides, our group [18] employed a charge-coupled device (CCD) with an image acquisition card as an image sensor to capture test strip images. However, the image acquisition limited the application of this instrument and, at the same time, resulted in complexity and high cost. In this article, an improved test strip reader is presented.

Gastric carcinoma is one of the common malignant tumors in the world [19]. Its morbidity and mortality, respectively, rank second and third among all malignant tumors. Nevertheless, only 10% or so patients were diagnosed with early gastric cancer (EGC) in China. Moreover, compared with ones suffering with late gastric cancer, patients with EGC have a higher survival rate [20], so early diagnosis of gastric carcinoma is of great importance. It is confirmed that *Helicobacter pylori* with cytotoxin-associated protein (CagA) is closely associated with gastric carcinoma's initiation and development [21-23]. If we could detect CagA as soon as possible, we might decrease or avoid development of gastric carcinoma via reasonable therapy. To realize this goal, we designed and prepared the device for ultrasensitive detection of CagA.

Herein, we reported that an improved CCD-based test strip reader was designed and developed. Besides, a corresponding software system was also developed for human-machine interaction. According to the CCD image sensor, test strip images were captured and then transmitted to the control computer. Afterward, the software system would finish the data analysis and present diagnostic results in the form of reports, which is a convenient diagnostic system for clinical physicians.

## Materials and methods

### Composition of test strips

**Figure 1:**
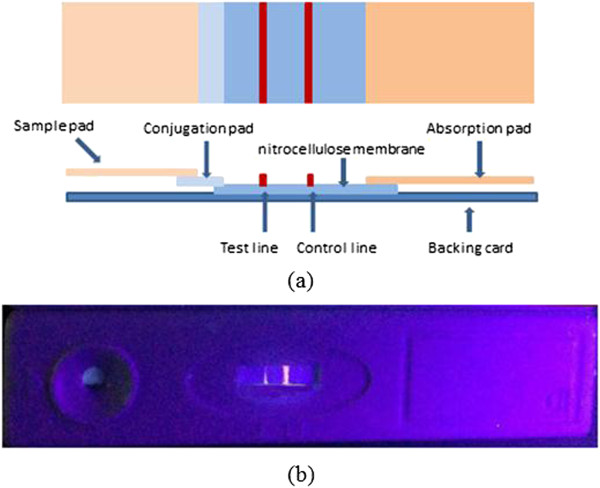
**Images of a test strip. (a)** Structure of a test strip. **(b)** Photo of a QD test strip under a UV lamp.

The immunochromatographic test strip (ITS) is composed of a sample pad, conjugation pad, nitrocellulose membrane, and absorption pad, as shown in Figure 1a. All these components are fixed onto a plastic backing card [5]. During the assay, the liquid sample is added onto the sample pad, and then the absorption pad wicks the liquid sample to the end of the test strip through capillarity. Analytes in the sample will combine with conjugates (labeled with CdSe quantum dots) in the conjugation pad. Subsequently, the formed complexes continue migrating along the membrane until they are captured by the test line (T-line). The residual will move forward and be captured in the control line (C-line). In this study, all the test strips were prepared by using the method reported by our group [7,18].

### Design of the hardware system

**Figure 2:**
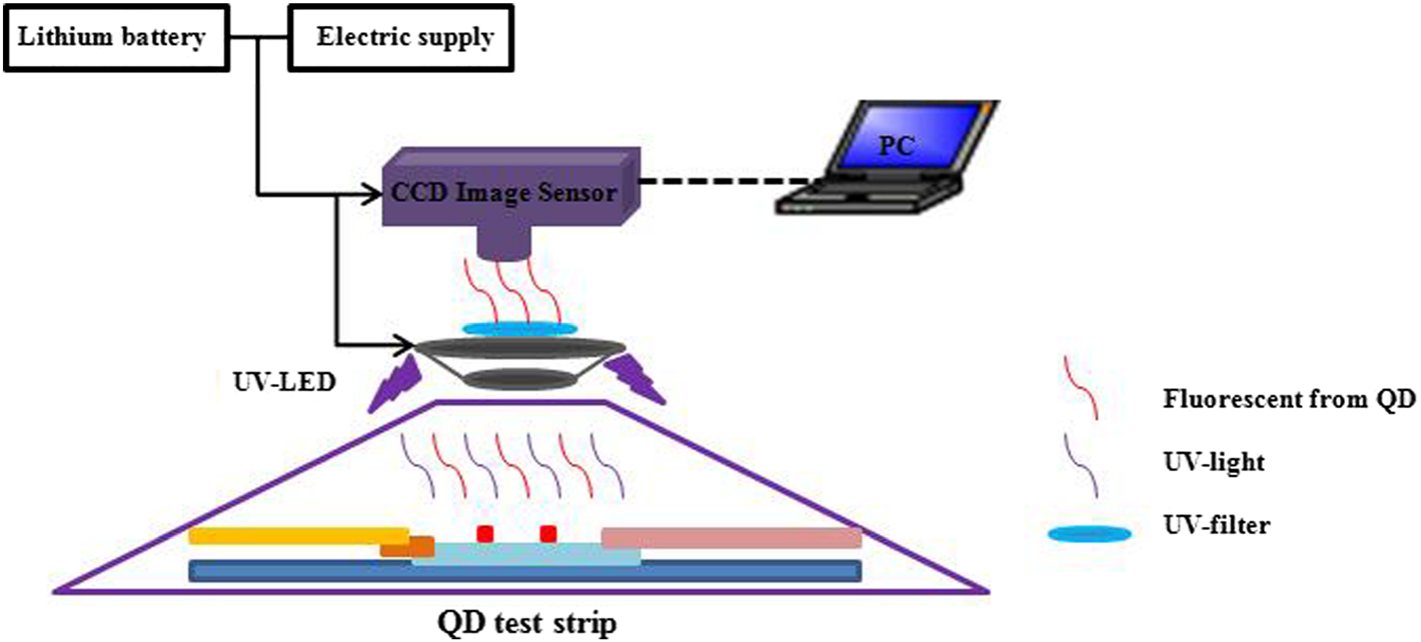
Structure of the CCD-based test strip reader.

The CCD-based test strip reader was composed of six modules, including a light source module, sample module, power module, acquisition module, radiator module, and PC module. The structure is displayed in Figure 2.

A quadrate ultraviolet LED as excitation light source was to make sure that samples accept the same amount of irradiation. It is also critical to select a good optical sensor. Photodiode, photomultiplier tube, linear CCDs, and image sensors are widely used optical sensors. However, photodiode, photomultiplier tube, and linear CCDs have a limited surveyed area. On the contrary, image sensors could provide a more flexible and wider detection area. Moreover, image sensors could realize short time detection [1]. Based on the above benefits, we decided to employ an image sensor. CCD and CMOS are two most popularly used image sensors. Compared with CMOS, CCD has the advantages of low noise and better imaging quality [24], so a color CCD image sensor was chosen. This digital CCD image sensor with a USB not only solved the problem of employing an image acquisition card but also provided stable and rapid data transmission.

The QD test strip was irradiated by an excitation light source and then produced fluorescence, which could be captured by the CCD image sensor. The captured image was transmitted to the computer and went through further processing to complete calculation of test results. In order to decrease background interference, an ultraviolet filter was added to resist the excitation light source. A lithium battery was adopted as power supply, providing a light source for places without electric supply.

### Development of the software system

**Figure 3:**
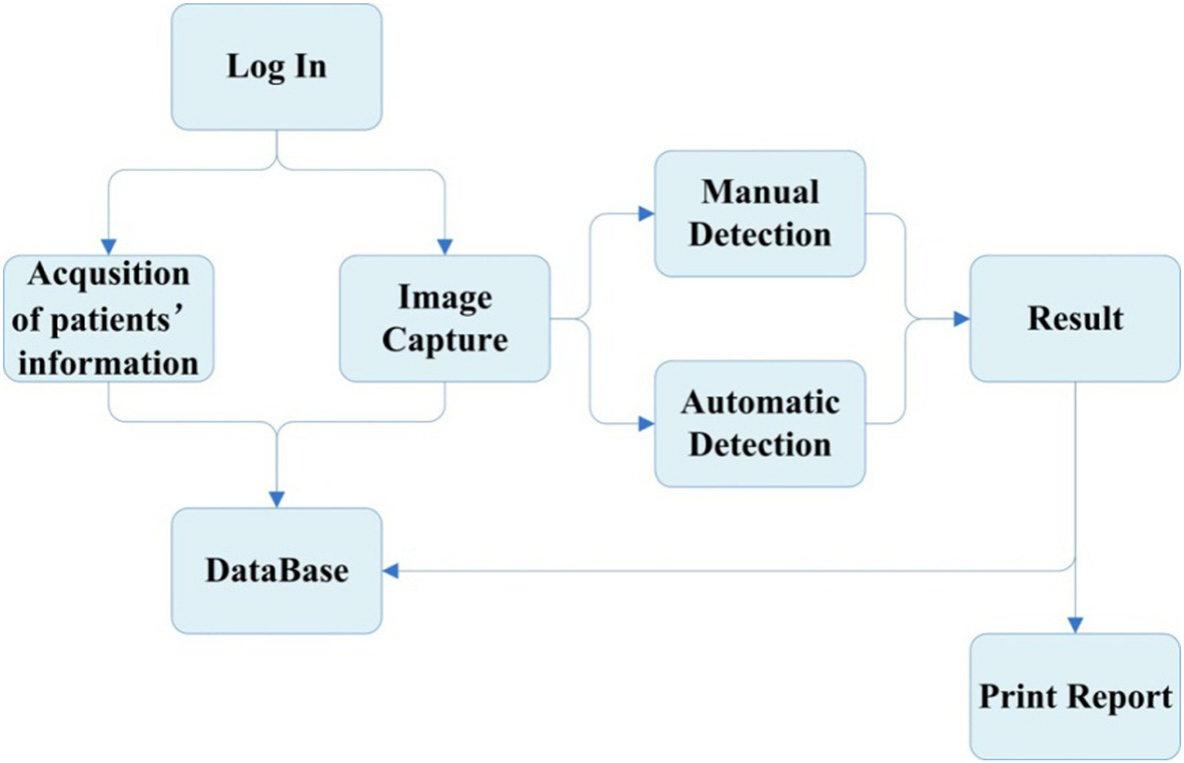
Flow diagram of the software system.

We also developed a suitable software system to give physicians easier access to our device. The software system was programmed in a Visual C++ development environment and provided main functions of processing test strip images, analysis, and diagnosis. Furthermore, the software system could be connected to a database and a printer for data storage or report printing. The flow diagram of the software system is shown in Figure 3.

In test strip images, the useful information was only T-line and C-line. However, there always existed intense background noise that requires to be separated. Therefore, an appropriate algorithm was proposed to reach this goal.

**Figure 4:**
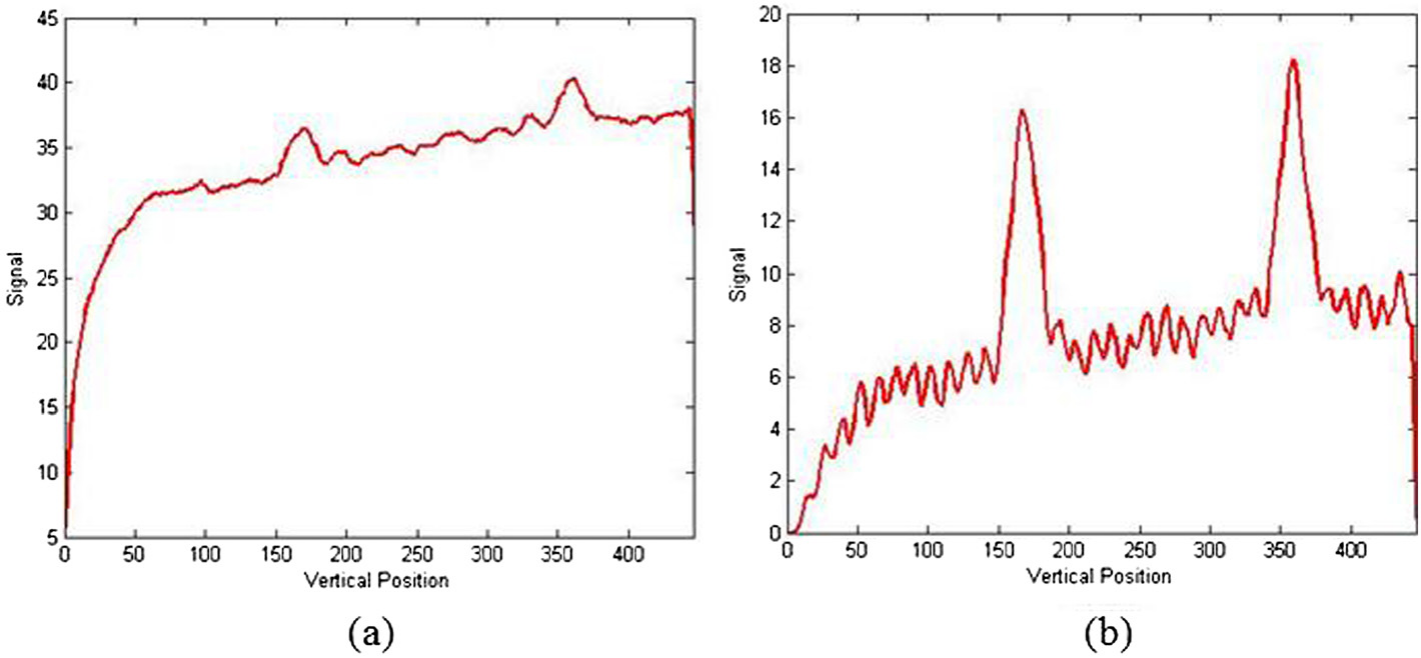
**Comparison between RGB and component R. (a)** Curve graph in RGB. **(b)** Curve graph in component R.

A revised weighted threshold histogram equalization (WTHE) algorithm was proposed. The WTHE contrast enhancement algorithm was first put forward by Qing and Ward [25]. In our study, this method was applied with some modification. By observation, the component R of red-green-blue (RGB) test strip images has an obvious difference between foreground and background. To indicate the advantage of component R, a comparison is shown. Figure 4 displays the results, from which it is obvious that the background in the component R image was lower than that in the RGB image. Hence, the mentioned contrast enhancement algorithm would be acted on the component R of test strip images. The procedure of the proposed algorithm is listed below.

Consider an image with *N* pixels and a gray level range of [0, *K* − 1].

1. Calculate the average gray value of all pixels named *T*. Then, scan all the pixels. These pixels’ value smaller than *T* will decrease a constant *C*.

2. Calculate the probability density function (PDF) *P*(*k*). *P*(*k*) = *n*_*k*_/*N*, *k* = 0,1…, *K* − 1, where *n*_*k*_ is the number of pixels with gray level *k*.

3. Compute an upper limit *P*_u_ and a lower limit *P*_l_ with great importance. *P*_u_ = *v* · *P*_max_, where *P*_max_ is the highest probability value and *v* represents the upper threshold normalized to *P*_max_ (*v* belongs between 0 and 1). *P*_l_ is a fixed value, which filters some very low probability values. Herein, *P*_l_ was set as 0.1%.

4. Define the new PDF. $P_{n}\left(k\right)=\left\{\begin{array}{c} P_{u},\;\mathrm{if}\;P\left(k\right)>P_{u}\\ {\left(\frac{P\left(k\right)-P_{l}}{P_{u}-P_{l}}\right)}^{r}\cdot \;P_{u},\;\mathrm{if}\;P_{l}\leq P\left(k\right)\leq P_{u}\\ 0,\;\mathrm{if}\;P\left(k\right)<P_{l} \end{array}\right)$. This step will remove very low probability pixels and limit very high probability pixels (background pixels).

5. Calculate the cumulative distribution function *C*_n_(*k*). $C_{n}\left(k\right)=\sum_{m=0}^{k}\;P_{n}\;\left(m\right)$.

6. Obtain the output image. *O*(*N*) = *n* · *W*_out_ · *C*_n_(*k*), where *W*_out_ is equal to the biggest value subtracting the smallest value and *n* represents the number of superposition.

## Discussion

### Characterization of CdSe QDs

**Figure 5:**
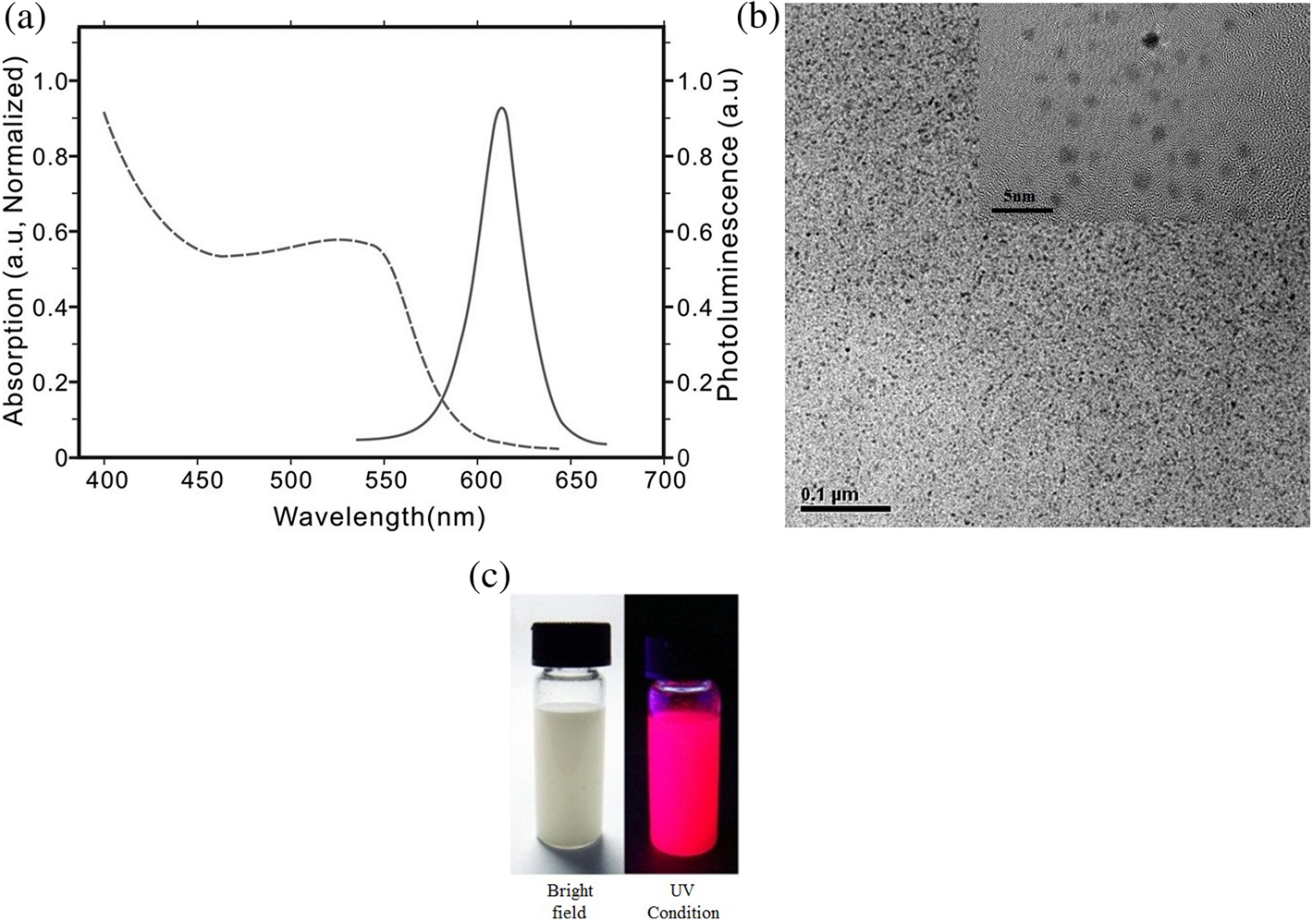
**Characterization of CdSe QDs. (a)** Absorption and emission spectrogram. **(b)** TEM picture of synthesized CdSe QDs. **(c)** Digital photos of the QD-labeled anti-CagA antibody before and after UV condition.

All the CdSe QDs were prepared by our group's member [7,18,26-29]. The absorption and emission spectrogram is displayed in Figure 5a. The emission wavelength was approximately 625 nm. The HR-TEM pictures (Figure 5b) show that the water-soluble CdSe QDs have a diameter of 5.4 nm. The digital photos of the QD-labeled anti-CagA antibody before and after UV condition are shown in Figure 5c.

### Hardware units

**Figure 6:**
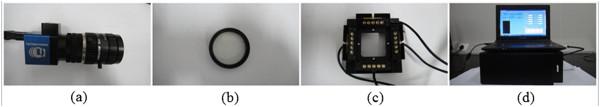
**Hardware units of the device. (a)** CCD image sensor. **(b)** UV filter. **(c)** Excitation light source. **(d)** Integrated instrument.

Figure 6 shows each component of the device. Figure 6a shows the CCD image sensor with a volume of 29 × 29 × 29 m^3^. Figure 6c,d represents the excitation light source and the integrated instrument, respectively. Compared with the use of the card acquisition card, that of the CCD image sensor with a USB is more convenient and has less cost. The UV filter is displayed in Figure 6b and could be connected with the CCD image sensor, playing an important role in eliminating interference of the light source. In addition, employing a lithium battery made the device viable without power supply for more than 6 h.

### Image processing results

**Figure 7:**
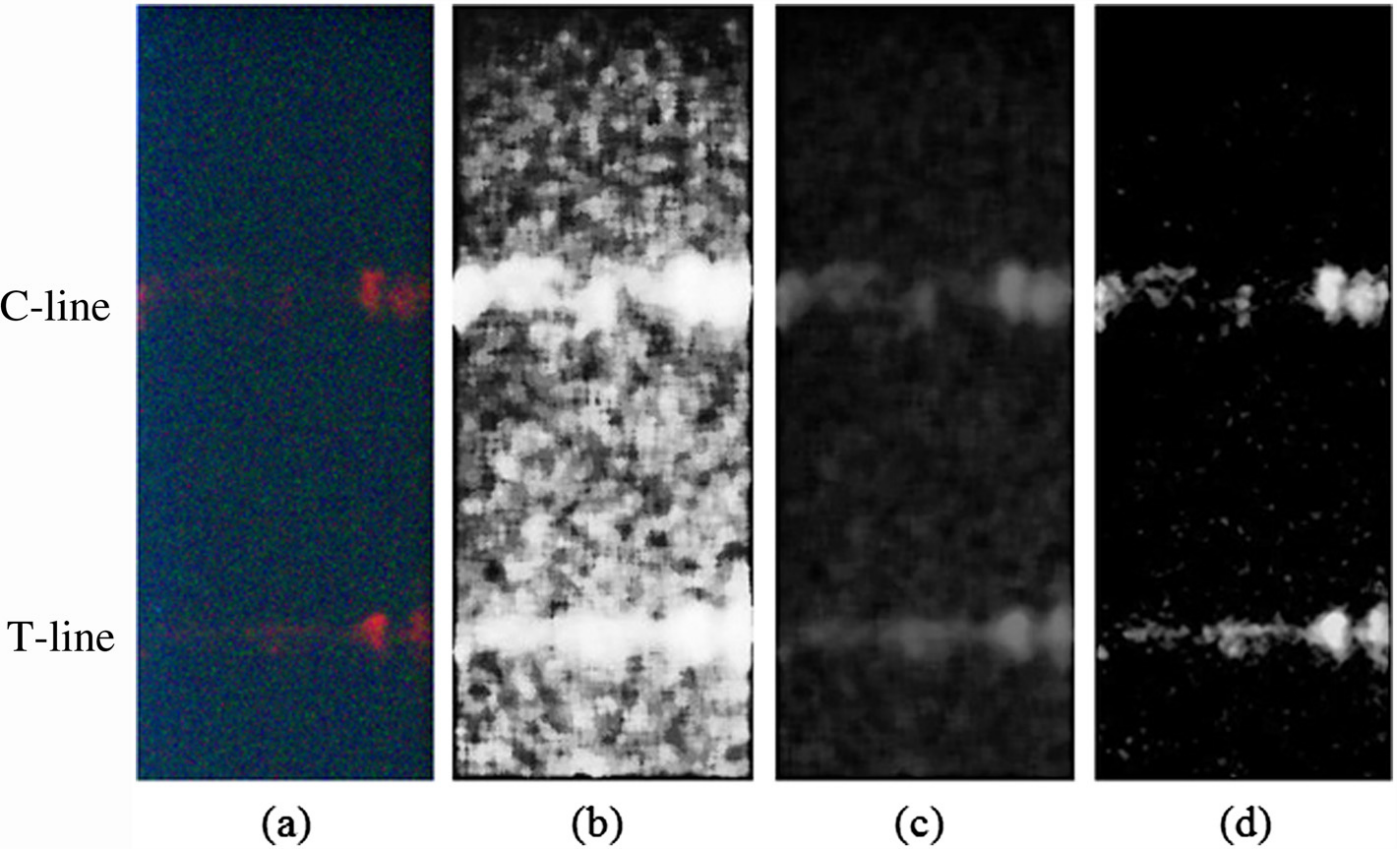
**Images of test strip. (a)** Original test strip image.**(b)** Test strip image with traditional HE. **(c)** Test strip image with WTHE. **(d)** Test strip image with proposed algorithm.

Figure 1b displays a photo of the QD test strip under the irradiation of ultraviolet light. Obviously, C-line and T-line emitted fluorescence. However, this photo was taken without a UV filter, containing a strong background caused by the excitation light source. As a comparison, Figure 5a presents a QD lateral flow strip picture with a UV filter, which accepted little influence of the excitation light source, demonstrating that the UV filter is essential. Though the effect of the excitation light source was eliminated, the fluorescent intensity was also weak in detecting a low-concentration sample (Figure 7a). The proper image enhancement algorithm played a critical role. In order to display our method's superiority, traditional HE and WTHE algorithms were compared with the proposed algorithm (Figure 7). Figure 7b,c shows test strip images after processing of the traditional HE and WTHE algorithms, respectively. The test strip image processed by the proposed modified WTHE algorithm is shown in Figure 7d. By comparing this method with other image enhancement algorithms, the results indicated that the proposed algorithm could produce a satisfying effect with distinguishing C-line and T-line from the background.

**Figure 8:**
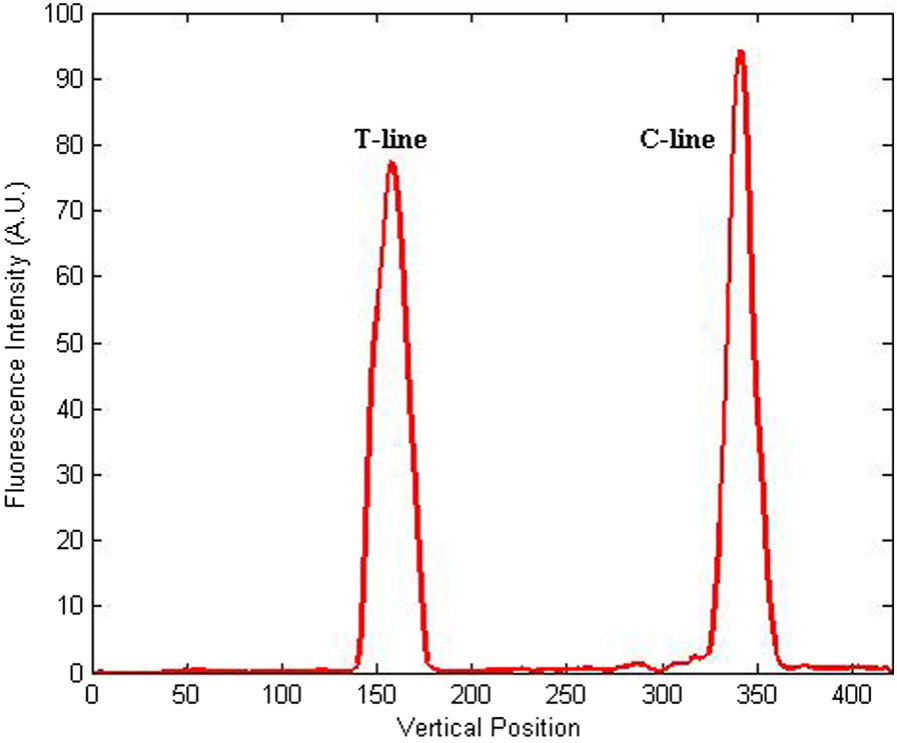
Curve figure of the test strip image after processing of the proposed modified WTHE algorithm.

During processing, we set *v* = 0.1 and *r* = 0.4, with the least mean square error (MSE). After image processing, the distinct graph of T-line and C-line is displayed in Figure 8 to certify the effectiveness of this algorithm.

### Diagnosis of CagA samples

In order to test the device, 50 positive and 50 negative CagA samples from a clinical hospital were collected for detection. The outcomes showed that the instrument could realize detection with a specificity of 98% and a sensitivity of 96%. The specificity and sensitivity are calculated according to the following equations, respectively:

$$\mathrm{Specificity}=\frac{\mathrm{True}\;\mathrm{negative}}{\mathrm{True}\;\mathrm{negative}+\mathrm{False}\;\mathrm{positive}}$$

$$\mathrm{Specificity}=\frac{\mathrm{True}\;\mathrm{negative}}{\mathrm{True}\;\mathrm{positive}+\mathrm{False}\;\mathrm{negative}}$$

**Figure 9:**
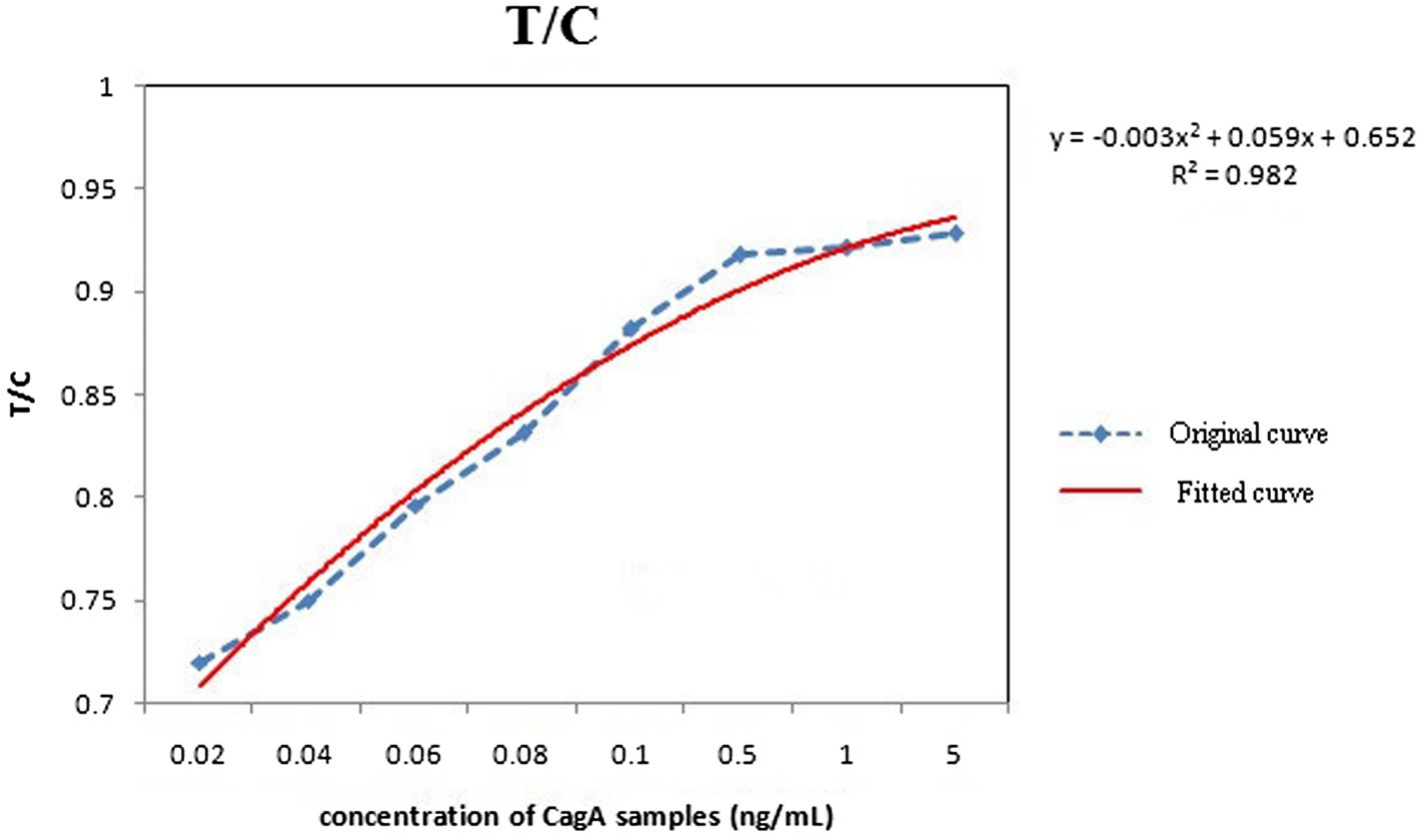
Graph of T/C in different concentrations.

Compared with naked eye detection, the device could recognize low-concentration samples by employing the proposed image processing algorithm, thus greatly enhancing sensitivity. Additionally, in order to improve specificity, more samples could be detected to set a more exact threshold. To eliminate the error introduced by the differences between test strips and samples, we used HCG samples to set a threshold of T/C ratio to determine the specification. The more antigen targeting in the sample, the more QD conjugates would be captured on the test line, which leads to the increase of the T/C ratio. According to the principle described above, the T/C ratio would be proportional to the concentration of CagA in the samples. Compared to the bare detection of the test line, this quantitative approach is much more credible and applicable. Besides, seven CagA samples with different concentration were also prepared by our group. From Figure 9, the relationship of T/C and concentrations could be represented by a fitting function. As the concentration increases, the value of T/C reaches the capacity. The device realized quantitative detection with a sensitivity of 20 pg/mL.

## Conclusions

In conclusion, a CCD-based reader was designed and fabricated, the quantitative analysis software was compiled, and the resultant CCD-based reader system was used for quantitative analysis of examined CagA antigen on the strips. A fluorescence detection system of lateral flow strip was developed. A revised WTHE algorithm was used to enhance captured QD test strip images. Practical results indicated that the system could quickly and accurately detect the fluorescence signal. QD lateral flow tests were used with different concentrations to detect CagA samples and indicated that the sensitivity of this device was 20 pg/mL. For a future study, test strips with multilines could be detected and some wireless technologies could also be applied in similar instruments. More nanoparticles could be applied for improving sensitivity, which is also a big issue.

## Competing interests

The authors declare that they have no competing interests.

## Authors’ contributions

CG, KW, XD, and DC carried out the development of the device. CL carried out the synthesis of CdSe QDs. All authors read and approved the final manuscript.

## Authors’ information

DC is a professor of Shanghai Jiao Tong University. His research interests include the synthesis of nanomaterials and their application in the biomedical field. KW is a lecturer of Shanghai Jiao Tong University. Her scientific interests are nanotechnology development of early cancer detection and screening equipment, nonmaterial molecular imaging, and biocompatibility evaluation. CL is a PhD candidate of Shanghai Jiao Tong University. XD and CG are both master students of Shanghai Jiao Tong University.
